# Radio-contrast agent-induced hyperthyroidism: case report and review of the literature

**DOI:** 10.1590/2359-3997000000143

**Published:** 2015-01-01

**Authors:** Ioannis Iakovou, Apostolos Zapandiotis, Vassileios Mpalaris, Dimitrios G. Goulis

**Affiliations:** 1 Third Department of Nuclear Medicine Medical School Aristotle University of Thessaloniki Greece Third Department of Nuclear Medicine, Medical School, Aristotle University of Thessaloniki, Greece; 2 First Department of Obstetrics and Gynecology Medical School Aristotle University of Thessaloniki Greece Unit of Reproductive Endocrinology, First Department of Obstetrics and Gynecology, Medical School, Aristotle University of Thessaloniki, Greece

## Abstract

A 66 year-old woman with a history of a euthyroid multinodular goiter underwent a head and neck computed tomography (CT) scan (total iodine load of 35 g) in order to evaluate the extent of retrosternal expansion. Less than 24 h after the iodine-based contrast media (ICM) administration, she presented with symptoms and laboratory findings typical of thyrotoxicosis. She was treated successfully with antithyroid medications. This is the shortest time reported in the literature and it is of clinical importance, as it may have an impact to the recommendations given by the attending physician. Given the fact that a large number of ICM examinations are performed in everyday practice, physicians should be aware of this possible thyroid-specific effect. Prophylactic drugs could be considered in high-risk populations, such as administration of perchlorate and a thionamide class drug to elderly patients with suppressed TSH and/or palpable goiter, started the day before and continued for two weeks after ICM administration.

## INTRODUCTION

Radiographic, water-soluble, iodine-based contrast media (ICM) solutions contain small amounts of free iodide, which may cause thyrotoxic crisis in patients with Graves’ disease or multinodular goiter and thyroid autonomy, especially if they are elderly and living in areas of iodine-deficiency (1). A recent review, summarizing the mechanisms of iodine-induced hypothyroidism and hyperthyroidism (2), identified ICM as an increasingly common source of supraphysiologic iodine exposure. Another review article recognized thyroid dysfunction as an important adverse outcome of ICM (3), suggesting that screening of the presence of risk factors before the use of ICM allow for early recognition of adverse reactions and prompt treatment. Finally, a recent study (4) discussed in detail the prevalence and types of ICM-induced thyroid dysfunction, indicating the populations that are at higher risk and summarizing the necessary prophylaxis and possible treatment.

The aim of this case report was to illustrate the clinical significance of ICM-induced thyrotoxicosis and to suggest prophylactic measures in patients at risk.

## CASE REPORT

**Figure 1:**
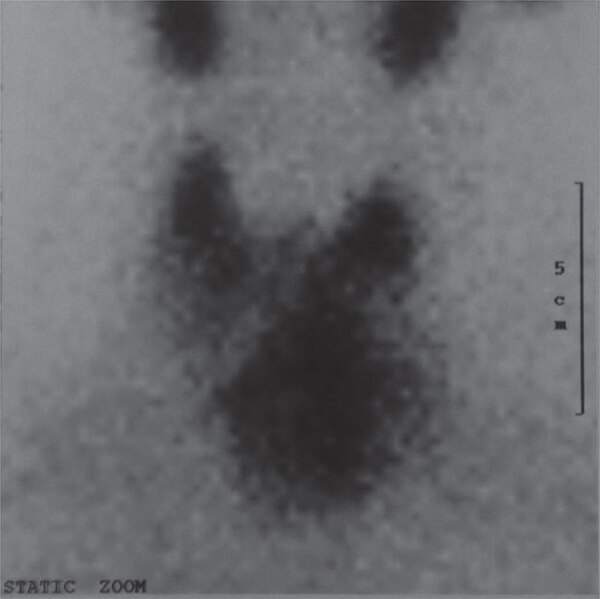
Pre-operative 99mTc scintigraphy (uptake 2.4%).

We present the case of a 66 year-old woman with a history of euthyroid multinodular goiter, submerging to the upper mediastinum. Serum thyroid stimulating hormone (TSH), free thyroxine (fT_4_) and free triiodothyronine (fT_3_) concentrations were normal at 1.8 mU/mL, 11.6 pg/mL and 2.9 pg/mL, respectively, with marginally positive thyroid autoantibodies [antibodies against thyroid peroxidase (anti-TPO) 75 U/mL, antibodies against thyroglobulin (anti-Tg) 120 U/mL, whereas antibodies against TSH receptor (anti-TSHR) were not measured]. Pre-operatively, a head and neck computed tomography (CT) scan followed the ^99m^Tc scintigraphy (uptake 2.4%), in order to evaluate the extent of goiter’s expansion and its anatomical limits (Figure 1). A volume of 100 mL of 350 mg I/mL contrast agent was administered intravenously giving a total iodine load of 35 g. Blood tests and ^99m^Tc-scintigraphy were performed one week before the CT scan took place. Less than 24 h after the CT, the patient complained of cardiovascular symptoms (tachycardia, increased blood pressure), sweat and tremor, suggestive of thyrotoxicosis. Laboratory findings were suggestive of hyperthyroidism (TSH 0.01 mU/mL, fT_4_ 29.6 pg/mL, fT_3_ 6.9 pg/mL, anti-TPO 215 U/mL, anti-Tg 360 U/mL). This ICM-induced thyrotoxicosis was improved clinically by conservative treatment with β-adrenergic blocking agent and thiamazole. Clinical improvement was achieved, specifically regulation of arterial pressure and tachycardia. Hormonal status improvement is not available as follow-up was not complete.

## DISCUSSION

Normally, iodine overload is accompanied by an auto-regulatory mechanism of inhibition of iodine organification in the thyroid gland, permitting regulation of hormone synthesis and secretion, in the presence of excessive amounts of iodine (Wolff – Chaikoff effect). Recommencement of normal organification, described as “escape phenomenon”, is assumed to occur a week to 10 days later, as a result of low inorganic iodine concentration, secondary to the down-regulation of sodium-iodide symporter (NIS) (5). In individuals with impaired auto-regulation, exposure to supraphysiological concentrations of iodine may lead to iodine-induced hyperthyroidism (Jod – Basedow phenomenon) (4). A dose of ICM used in a typical imaging procedure has a volume between 100 and 200 mL and contains about 13,500 μg of iodide and 15 – 60 g of bound iodine that may be liberated as free iodide. This is actually an acute iodide load of 90 to several hundred thousand times the recommended daily intake of iodide (150 μg).

The prevalence ICM-induced thyroid disease has not been assessed accurately (6), varying widely from 0.05% to 5% (4), and being greater among patients with pre-existing thyroid disease. The odds ratio (OR) for developing hyperthyroidism has been estimated to 1.98 [95% confidence interval (CI) 1.08 - 3.06] (6). Risk factor for the pathogenesis of this phenomenon is every condition with thyroid autonomy, such as Graves’ disease or, as in our case, multinodular goiter (7,8). The presence of autonomous thyroid function is supposed to permit the synthesis and release of excess quantities of thyroid hormones. Clinical manifestations cannot be differentiated from other forms of thyrotoxicosis. Thyroid dysfunction may be subclinical or overt. The timing of onset may be up to 12 weeks after ICM administration.

Little is known about the short-term effects on thyroid function of supramaximal doses of iodine in euthyroid patients. It has been suggested (9) that the application of high amounts of ICM might induce transient subclinical hypothyroidism within the first week, even in patients with no apparent thyroid abnormality. Patients with basal TSH concentrations above 2 μU/mL are at high risk (9). In our case, there was an extremely short time interval (< 24 h) between ICM administration and onset of symptoms. According to our knowledge, this is the shortest time reported in the literature and it is of clinical importance, as it may have an impact to the recommendations given by the attending physician: the patient has to be informed that signs and symptoms of hyperthyroidism may occur any time during the 12 weeks following ICM administration.

Prevention of iodine-induced thyrotoxicosis in high-risk patients is important, as treatment with thyrostatic drugs is hindered by the high iodide concentrations and complications are more frequent than in other forms of thyrotoxicosis (1). In cases of established hyperthyroidism, administration of ICM is contra-indicated. To reduce the incidence of iodine-induced thyrotoxicosis, it has been suggested that prophylactic drugs could be administered, starting well before the examination. Administration of perchlorate and a thionamide class drug to elderly patients with suppressed TSH and/or palpable goiter has been recommended, started the day before and continued for two weeks after ICM administration. The following regimen has been suggested: thiamazole 30 mg once daily, starting the day before the exam (or directly prior to exam, in case of emergency) that has to be continued for 14 days (1). We believe that universal prophylactic treatment should not be administered, especially in elderly patients; nevertheless, these patients need to be followed carefully.

Given the fact that a really large number of ICM examinations are performed in everyday practice, the uncommonly adverse events of iodide overload will consequence a considerable number of patients being affected. Thus, physicians should be aware of this possible thyroid-specific effect (4).
